# Natural products provide a new perspective for anti-complement treatment of severe COVID-19: a review

**DOI:** 10.1186/s13020-021-00478-3

**Published:** 2021-07-28

**Authors:** Yadong Fan, Ying Wang, Shuang Yu, Jun Chang, Yiqi Yan, Yiyang Wang, Yuhong Bian

**Affiliations:** 1 School of Integrative Medicine, Tianjin University of Traditional Chinese Medicine, No.10 PoYangHu Road, JingHai, District, Tianjin, 301617 People’s Republic of China; 2 Institute of Traditional Chinese Medicine, Tianjin University of Traditional Chinese Medicine, Tianjin, China

**Keywords:** Natural products, Cytokine storm, COVID-19, Critically ill, Anti-complement therapy

## Abstract

Exaggerated immune response and cytokine storm are accounted for the severity of COVID-19, including organ dysfunction, especially progressive respiratory failure and generalized coagulopathy. Uncontrolled activation of complement contributes to acute and chronic inflammation, the generation of cytokine storm, intravascular coagulation and cell/tissue damage, which may be a favorable target for the treatment of multiple organ failure and reduction of mortality in critically ill patients with COVID-19. Cytokine storm suppression therapy can alleviate the symptoms of critically ill patients to some extent, but as a remedial etiological measure, its long-term efficacy is still questionable. Anti-complement therapy has undoubtedly become an important hotspot in the upstream regulation of cytokine storm. However, chemosynthetic complement inhibitors are expensive, and their drug resistance and long-term side effects require further investigation. New complement inhibitors with high efficiency and low toxicity can be obtained from natural products at low development cost. This paper puts forward some insights of the development of natural anti-complement products in traditional Chinese medicine, that may provide a bright perspective for suppressing cytokine storm in critically ill patients with COVID-19.

**Supplementary Information:**

The online version contains supplementary material available at 10.1186/s13020-021-00478-3.

## Background

### Epidemiology and Pathogenesis of SARS-CoV-2 infection

In the first 20 years of the twenty-first century, we have witnessed the rapid spread of severe acute respiratory syndrome (SARS), Middle East respiratory syndrome (MERS) and severe coronavirus disease 2019 (COVID-19) around the world, posing an unprecedented threat to the world's public health system and attracting worldwide attention. As of July 2021, an outbreak of COVID-19 caused by severe acute respiratory syndrome coronavirus 2 (SARS-CoV-2) has affected 215 countries and regions, with more than 184 million reported cases and 3.9 million deaths according to statistics from World Health Organization and national notifications. Organ dysfunction, especially progressive respiratory failure and generalized coagulopathy are associated with maximum mortality [1–3].

During the SARS outbreak in 2003, Angiotensin-converting enzyme 2 (ACE2) was identified as a functional receptor for SARS-CoV, which mediates viral invasion into host cells [4]. ACE2 consists a C-terminal collectrin-like domain, an N-terminal peptidase domain and a transmembrane helix [5]. The spike protein (S protein) on the surface of CoV is a key protein that determines the transmission ability and host range of virus. The S protein has two subunits with different functions. The S1 subunit is responsible for receptor binding including the C-terminal receptor binding domain and N-terminal domain, while the S2 subunit drives fusion of viral and host cell membranes containing the transmembrane domains and fusion peptide [6, 7]. The receptor binding domain of S1 subunit interacts with peptidase domain of ACE2 to form a virion-ACE2 complex, which is then transported to enter the endosome of host cells [8]. ADAM17 is a member of the metalloproteinase family, which can cleave membrane-bound ACE2 and release its extracellular part into the circulation as soluble ACE2 (sACE2). Although sACE2 lacks transmembrane and cytoplasmic domains, its activity is retained [9]. In vitro studies have shown that SARS-CoV replication can be attenuated by ADAM17 inhibitors [10]. The S protein also forms a six-helix bundle core to promote fusion of cellular membrane with viral envelope. Finally, the viral RNAs are released into the cytoplasm of the target cells. Subsequently, the SARS-CoV-2 RNA genome interacts with the viral RNA replicase and transcriptase in a complex [11, 12]. During this period, the translated structural proteins and cytoplasmic virus RNAs assemble into new viruses, which are released from host cells and invade other susceptible target cells continuously.

SARS-CoV-2 and SARS-CoV share homology and similarity in their genome sequences [13]. The homology modeling also shows that the receptor-binding domains of the two are similar [14, 15]. Recent studies have supported that SARS-CoV-2 likes SARS-CoV infecting host cells through the combination of S protein and ACE2 on the host cell membrane surface [16–18]. ACE2 is widely distributed in lung alveolar cells, kidneys, heart, intestines, endothelium, mouth, brain and testes, which may explain the impact of SARS-CoV-2 on lung injury, acute kidney, cardiac damage, gastrointestinal symptoms and potentially cardiorespiratory depression [19]. Apart from attachment, SARS-CoV-2 entries into host cells is determined by S protein cleavage at two proteolytic cleavage sites, named S1/S2 and S2’ subunits, which are processed by the cellular protease furin and serine protease transmembrane protein serine protease 2 (TMPRSS2) respectively [20]. Compared with SARS-CoV, there is a polybasic furan cleavage site in the S protein of SARS-CoV-2 [21]. Furin-like proteases may contribute to the more general expression and processing of S protein, that explains the expanded cell and tissue tropism of SARS-CoV-2 [22]. The lung and intestines express ACE2 and TMPRSS2, which are the main invasion sites of SARS-CoV-2 [23]. The body's immune response to SARS-CoV-2 and mechanism of immune pathological changes have been reported in detail in the literature [24].

## Cytokine storm accounts for the severity of COVID-19

**Fig. 1:**
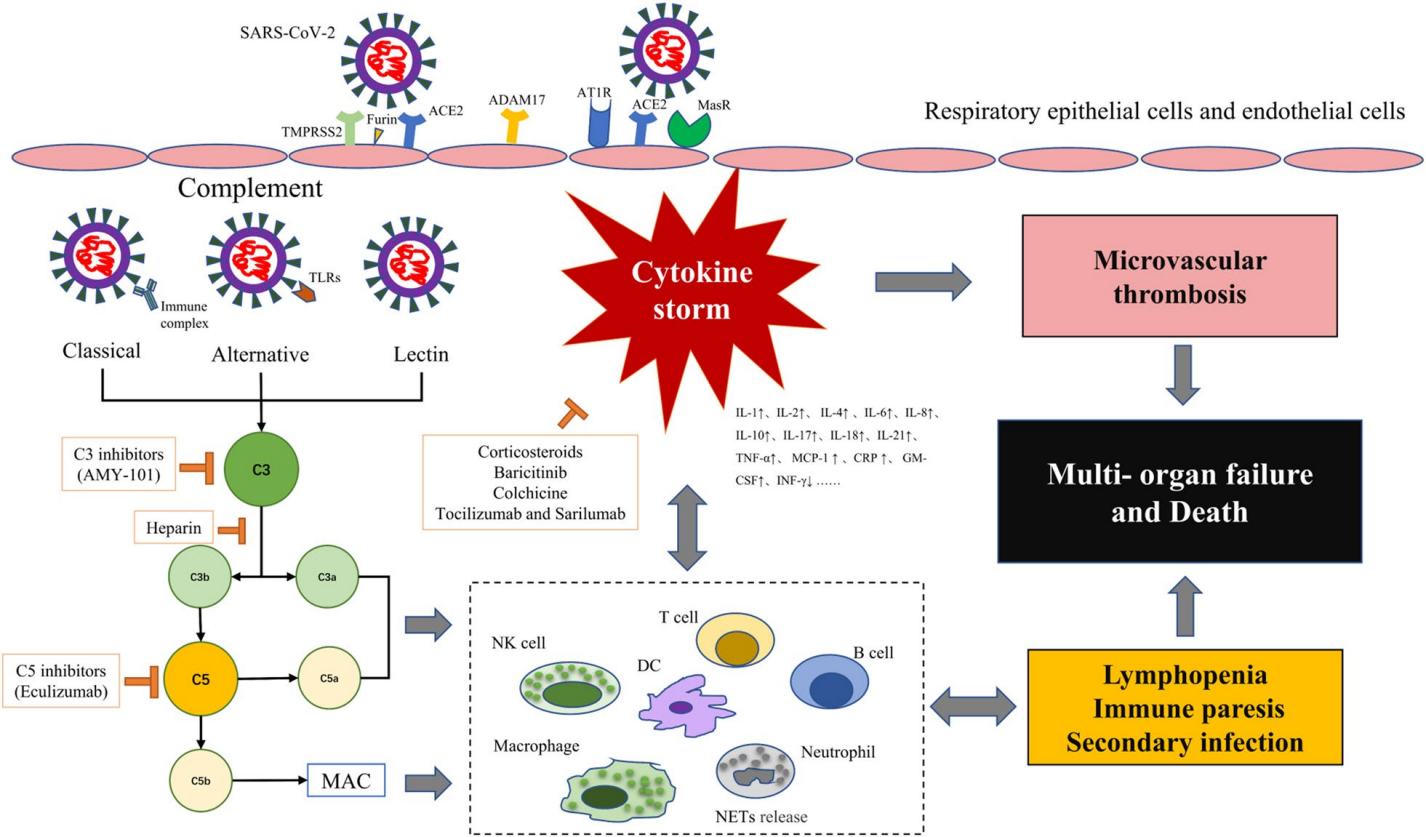
Abnormal activation of complement interacts with pro-inflammatory cells, cytokines, coagulation and thrombotic microangiopathy mechanisms during the SARS-CoV-2 infection. The abnormal activation of complement after SARS-CoV-2 infection interacts with pro-inflammatory cells, cytokines, coagulation and thrombotic microangiopathy mechanisms, leading to the generation of cytokine storms. Cytokine storms cause lymphopenia, immune paresis, secondary infection, multiple organ failure and even death, which are more prominent in critically ill patients with COVID-19

The progression of SARS-CoV-2 infection can be divided into four phases, including upper and lower respiratory tract infection, lung injury associated with COVID-19, systemic inflammatory response syndrome and systemic failure (local infections progress into systemic pathology) [25]. The immune response is critical to the resolution and control of viral infection, but it can also result in immunopathogenesis, related to the uncontrolled immune response [26]. Pathological and observational studies of COVID-19 patients have shown the systemic cytokine effects of infection (also known as cytokine storm), accompanied by an increase of related inflammatory clinical markers, and a procoagulant, thrombotic milieu (Fig. 1) [27–30]. The development of the cytokine storm is characterized by the rapid hyperactivation and proliferation of white blood cells including natural killer cells (NK cells), macrophages, dendritic cells (DCs), T and B lymphocytes (T and B cells), and the aberrant release of more than 150 chemical mediators and inflammatory cytokines [26, 31, 32]. Uncontrolled overproduction of inflammatory cytokines leads to multi-organ damage like acute respiratory distress syndrome (ARDS), gastrointestinal tract dysfunction, coagulopathy, cardiac injury, kidney dysfunction and neurological impairment, which are often demonstrated in severe and critically ill patients associated with SARS-CoV-2 infection [33–36]. Based on the pathogenesis, pathology and clinical features of complications induced by SARS-CoV-2, the evidence suggests that exaggerated immune response and cytokine storm account for the disease’s severity [37, 38].

For patients with COVID-19 in the intensive care unit (ICU), the number of neutrophils, white blood cells, and levels of C-reactive protein, procalcitonin, as well as other inflammatory indices, are significantly higher than those in non-ICU [2, 30]. It is worth noting that the plasma levels of granulocytes colony stimulating factor (GCSF), macrophage inflammatory protein-1A, interferon-γ-induced protein 10 (IP-10), monocyte chemoattractant protein 1 (MCP-1), interleukins IL-2, IL-6, IL-7, IL-10 and tumor necrosis factor-α (TNF-α) are significantly elevated in ICU patients, confirming the cytokine storm related to the severity of the COVID-19 disease [39]. SARS-CoV-2 infection and rapid replication lead to vascular leakage and the apoptosis of a large number of lung epithelial cells and endothelial cells, resulting in increased release of pro-inflammatory cytokines and chemokines, including IFN-γ, IP-10, MCP-1, IL-1, IL-4 and IL-10, that destroys the barrier of pulmonary microvascular and alveolar epithelial cells, leading to ARDS [27, 40]. When the S protein of SARS-CoV-2 attaches to ACE2, its intracellular binding site down-regulates ACE2, which may cause the cytokine storm through dysregulation of ACE/angiotensin II/AT1R axis, attenuation of ACE2/MasR axis and increased activation of ACE2/bradykinin B1R/DABK axis [41]. The storm also attracts massive neutrophils and monocytes, resulting in excessive inflammatory cells entering into lung tissue. Upstream of the cytokine storm, SARS-CoV-2 first induces IL-1 and TNF-α production of monocytes that act on stromal cells inducing the production of IL-6, IL-8 and granulocyte–macrophage colony-stimulating factor (GM-CSF) to affect neutrophils [42]. IL-6/8 are key chemokines that promote the activation and migration of neutrophils. IL-6 forms a complex with membrane IL-6 receptors on the surface of neutrophils or soluble IL-6 receptors to act on gp130, regulating levels of GM-CSF, MCP-1 and IL-6 through the Janus kinase-signal transduction and transcription activator (JAK-STAT) pathway, thereby prolonging the inflammatory processes [43]. A similar signal transduction occurs after IL-8 binds to CXCR1/2 on the surface of the neutrophils. Those effects are significantly enhanced under the synergistic effect of IL-17 produced by Th17 or other IL-17-producing cells. The analysis of wide genome screening and quantitative proteomics of peripheral blood mononuclear cells in COVID-19 revealed that non-structural protein 9 and 10 (nsp9 and nsp10) target NF-κB-repressing factor to facilitate IL-6/IL-8 induction, which may contribute to the chemotaxis of neutrophils and excessive host inflammatory response [44]. In addition, together with other pleiotropic cytokines, IL-6 promotes the acute phase response and increases CRP, serum ferritin, complement, and procoagulant factors. Soon after, a vicious circle is formed which promotes the loss of regulation of the innate and adaptive immune response, and the cytokine storm, coagulation and thrombosis are further activated, leading to severe systemic tissue damage, organ failure, and even death [45, 46]. The report showed that severe COVID-19 patients often suffer from acute kidney injury, liver dysfunction and heart injury [47]. A large number of patients die due to improper management of the late stage of the disease.

Lymphopenia is a significant sign of COVID-19, which is also a diagnostic standard for COVID-19 in China [48]. Previous studies have shown that patients with severe COVID-19 have acute lymphopenia and damage to lymphatic tissues such as the spleen and lymph nodes, leading to immune paralysis that interferes with the clearance of virus [49]. The enhanced ratio of neutrophils to lymphocytes was observed in severe COVID-19 patients with lymphopenia, which is considered a clear sign of systemic inflammation and poor prognosis [50]. In COVID-19 patients, the T cell and NK cell counts are reduced, and significantly lower in the critically ill cases [51–53]. The lung autopsy pathology of the deceased from COVID-19 showed the presence of ARDS and excessive activation of T cells, presumably caused by the high cytotoxicity of CD8^+^ T cells and an increase in the number of CCR4^+^ CCR6^+^ T-helper 17 cells [54]. SARS-CoV-2 can lead to the exhaustion of NK cells and CD8^+^ T cells by up-regulating the exhaustion markers like NKG2, and it also causes activation-induced cell death of CD169^+^ macrophages through Fas/FasL interactions [51]. Transcriptomics indicated that SARS-CoV-2 could also activate the P53 signaling pathway and apoptosis of lymphocyte, which partly explains the cause of lymphopenia [55]. It is worth noting that immune-histochemical staining showed CD4^+^ T cells and CD8^+^ T cells were reduced, which was related to an overexpression of proinflammatory chemokines and cytokines [49]. However, the overall mechanism of lymphocytopenia of critically ill COVID-19 patients in a state of cytokine storm is not conclusive, given that ACE2 receptors have not been found on lymphocytes, the destruction of lymphocytes is only theoretically due to cytokine storm [48].

For now, many laboratory findings emphasize the vital role of systemic inflammation downstream of viral infection, and the conversion of infectious diseases. Steroid intervention can prevent COVID-19 progression, which indicates that the pathophysiology of COVID-19 is mediated by viral infections, but also the result of the host inflammatory immune response [40, 56]. In addition, the fatal killer of H1N1, H5N1 and H7N9, as well as Ebola virus, is also proven to be a cytokine storm, which triggers the immune system to violently attack the body's tissues and organs [57–59].

## Treatment of cytokine storm

On January 30th, 2020, the first guidelines of COVID-19 diagnosis and treatment were released and monitoring of cytokines to improve the cure rate and reduce mortality was recommended [60]. Immunosuppression is likely to be beneficial when hyper-inflammation occurs later in patients with severe viral infections. Identification and treatment of cytokine storm is likely to be beneficial to address the immediate need to reduce the rising mortality in patients with severe viral infections [27]. Relative therapeutic options include a variety of nonspecific immunosuppressive strategies, such as glucocorticoids, JAK inhibition, colchicine, as well as hydroxychloroquine, and selective cytokine blockade such as anti-IL-6 (Tocilizumab and Sarilumab) and anti-IL-1 (Anakinra) [27, 61].

Corticosteroids with powerful anti-inflammatory and anti-fibrotic properties, and theoretically have the effect of inhibiting lung inflammation. They have been widely used in critically ill patients with SARS and MERS. Glucocorticoids can reduce the proliferation, differentiation, activation and survival of T cells and macrophages, and inhibit the synthesis of downstream proinflammatory cytokines TNF-α, IL-1, IL-2, IL-6, IL-17, GM-CSF and inducible cyclooxygenase-2 of Th1 cells and macrophages, through inducing lipocortin-1 and inhibition of NF-κB signaling [51]. Based on existing research and clinical experience, the potential benefit of low-dose corticosteroid therapy for critically ill patients with COVID-19 is recognized, although the overall survival rate has not been significantly improved [62]. Considering the clear advantages of glucocorticoids in terms of global availability and cost, it remains a key first-line choice of ICU patients infected with SARS-CoV-2 [2, 63, 64]. According to the previous treatment results and evidence of SARS and MERS, corticosteroids can delay the clearance of coronavirus and increase the mortality of virus infection [64]. Furthermore, glucocorticoid-mediated stimulation of the hypothalamic–pituitary–adrenal axis may also aggravate lymphopenia [65]. In view of the negative clinical results, corticosteroids are not recommended in routine use of COVID-19 treatment [66]. Ultimately, using corticosteroids to treat COVID-19 is a double-edged sword. But for critically ill patients, the timing, dose, and duration of the corticosteroid therapy remains worthy of further research and clarification.

Baricitinib, a JAK1 and JAK2 inhibitor, is predicted to reduce SARS-CoV-2 entry and inflammation of COVID-19 patients, which is suggested to combine with antiviral therapy in severe cases [67, 68]. However, the inhibition of IFN production and the damage of antiviral immune function caused by the blocking of the JAK-STAT pathway may limit its use [69]. Colchicine can inhibit IL-1β secretion and neutrophil recruitment to the site of inflammation, and its trial for COVID-19 is in progress (NCT04322682 NCT04322565, NCT04328480 and NCT04326790). A retrospective study found that colchicine may not protect patients against SARS-CoV-2 infection [70]. The safety of colchicine in clinical use has also been questioned [71]. Unfortunately, a recent randomized trial confirmed that hydroxychloroquine, as post-exposure prophylaxis for COVID-19, did not provide a significant benefit to prevent confirmed infection or illness compatible with COVID-19 [72].

Tocilizumab, a recombinant human IL-6 monoclonal antibody, can improve fever, oxygenation, pulmonary lesions, CRP levels and the percentage of peripheral lymphocytes in critically ill COVID-19 patients [73]. Another anti-IL-6 monoclonal antibody, Sarilumab, was received approval to a clinical trial and the efficacy and safety of anti-IL-1 antibody Anakinra in COVID-19 patients is carrying out (NCT04324021).

Ultimately, in the application of drugs related to suppressing cytokine storm, the balance between beneficial and harmful effects, the correct time frame and dosage of use should be clearly specified and considered. Furthermore, cytokine storm inhibition is only used as a remedial etiological measure, manifesting some relief of clinical symptoms in severe patients, but the long-term effectiveness remains questionable. Given the abundance and variety of cytokines in critically ill patients, more consideration should be given to effective regulation from the upstream stage of cytokine storm, which may have more application value.

## Complement as a target of inhibiting cytokine storm in COVID-19

The complement system as the early innate immune responses of the host immune system to pathogens, its uncontrolled activation contributes to acute and chronic inflammation, the generation of cytokine storm, intravascular coagulation and cell/tissue damage, which ultimately leads to multiple organ failure, and even death [74, 75]. The complement system comprises a network of membranes and soluble proteins that act in a coordinated manner on the activation of classical pathway (CP), lectin pathway (LP), and alternative pathway (AP) [76]. The complement cascade is helpful in controlling the initial viral infection through virus uptake and removal by phagocytes, coating virions to prevent them from attaching to their receptor, lysis of virus by pore formation, and destruction of its cell membrane by forming membrane attack complex (MAC, or C5b-9) [77]. As the infection continues to occur, due to the direct over-activation of virus or damaged host tissues, complement may become harmful. Genetic mutations or protein deficiencies inevitably give rise to insufficient or excessive complement activation, which triggers and/or maintains various pathological conditions like acute and/or chronic inflammation, disseminated intravascular coagulation (DIC), and even multiple organ failure and death [76, 78].

Immuno-histochemical analysis of lung tissue in death of patients with COVID-19 showed that complement components mannan-binding lectin (MBL), C3, C4 and C5b-9 in exudates of alveolar spaces, some pneumocytes, alveolar epithelial cells and inflammatory cells were strongly stained. Severe COVID-19 patients have higher serum complement protein C5a level than mild patients and healthy people. The CoVs’ nuclear proteins bind to mannan-binding lectin serine protease 2 (MASP-2), a key protein of the LP activation, resulting and aggravating inflammatory lung injury [79, 80]. In addition, C5b-9, C4d and MASP-2 deposits were found in the microvasculature of lung and skin biopsy in severe COVID-19 patients, consistent with activation of the AP and LP of complement [81]. Complement activation may also contribute to microvascular endothelial cell injury and the clotting pathway activation mediated by C5b-9, leading to fibrin deposition [82]. Indeed, the very high d-dimer levels were found in three critically ill cases. According to the analysis of autoptic kidney tissues, SARS-CoV-2 not only directly infects human kidney tubules, but also initiates CD68^+^ macrophages together with complement C5b-9 deposition to mediate tubular pathogenesis. In the transcriptomics of peripheral blood mononuclear cells of COVID-19 patients, up-regulated genes were significantly enriched in complement activation and classical pathways [83]. In summary, the complement in the circulation, lungs and kidneys of COVID-19 patients is strongly activated.

Complement activation, particularly C3a and C5a, has been widely demonstrated as involved in the development of acute lung damage induced by pathogenic viruses. C3a and C5a have intrinsic pro-inflammatory activities in activation and trafficking of immune cells (e.g., T cells, B cells, neutrophils and macrophages) and synergize with some innate immune sensors, such as Toll-like receptors (TLR2, TLR4 and TLR9), to amplify inflammation (e.g., TNF-α, IL-1, IL-6 and IL-8), which might be the main contributors of cytokine storm [84–86]. After influenza virus infection, the expression of C5aR on DC cells increases, which can promote the activation of CD8^+^ T cells by C5a [87, 88]. C5a also activates macrophages and endothelial cells to promote blood vessel leakage and the release of oxidants and enzymes [78, 89]. The activated complement cascade produces C3b, C5b fragments and C5b-9 that can induce the synthesis of arachidonic acid metabolites, including leukotrienes, thromboxane B2 and prostaglandin E2, to further recruit and activate neutrophils, monocytes and eosinophils, and stimulate the production of pro-inflammatory cytokines and mediators [90, 91].

Neutrophil extracellular traps (NETs), as a kind of innate immune effector defense mechanism, are the extracellular webs containing histone proteins, DNA, oxidant enzymes and some other microbicidal/nuclear proteins released by neutrophils [92]. In an influenza outbreak, C5a can activate neutrophils to release reactive oxygen species, inducing a wide variety of cell apoptosis, cell necrosis and cytokine release. C5a can also stimulate excessive NETs production, resulting in collateral epithelium and alveolar damage, and increased alveolar capillary barrier permeability [93, 94]. A recent study reported for the first time that many hospitalized COVID-19 patients have elevated serum NETs, providing evidence that patients are in a pro-NETotic state [95]. In fact, excessive NETs formation can trigger a series of inflammatory reactions, destroy surrounding tissues, promote DIC, and cause permanent organ damage to the lung, renal, and cardiovascular systems. Coincidentally and importantly, those are also three affected organ systems commonly seen in severe COVID-19 [96–98]. Both complement and neutrophils are key sentinels of innate immunity and regulate the pathway of thrombosis. The complement is thought to be cross-related with neutrophil-mediated C5a receptor/tissue factor [99]. The endogenous and exogenous coagulation pathways interact with complement through various two-way interactions to help maintain the steady state of coagulation and fibrinolysis. A recent review summarized several mechanisms by which SARS-CoV-2 infections activate coagulation and thrombotic microangiopathies by interacting with complement, pro-inflammatory cells and cytokines [100]. The first step in the activation of LP is a complex of MBL with MASP-2, which is part of the positive feedback loop that leads to sustained AP activation, accompanied by activation of inflammation and coagulation cascades [101, 102]. A number of studies have confirmed the role of AP in DIC, characterized by C5b-9 deposition [82, 103]. Pathogenic viral infection disrupts this balance and forms an overall thrombotic inflammation state, which is also reflected in critically ill COVID-19 patients (Fig. 1).

C5a can also mediate immune paresis playing a key role in ALI of patients infected with H7N9, SARS-CoV-1, MERS-CoV and SARS-CoV-2 viruses [100, 104]. C5a induces apoptosis of thymocytes, leading to overall reduction of T cells. In SARS-CoV-1 and MERS-CoV infections, C5a induces the release of IL-6 and IL-8 to further reduce the antigen presentation ability and capacity to produce antiviral cytokines such as IFN-α, IFN -β, IFN -γ and IL-12 of DCs [104, 105]. Indeed, elevated plasma C5a levels in COVID-19 patients are consistent with the clear role of C5a in promoting lung sequestration of leukocytes and pulmonary dysfunction, that reflects the severity of the disease. It also indicates that sC5b-9 has similar effects, which can cause leukocyte migration across the epithelium and vascular leakage [80, 106].

## Anti-complement therapy for critically ill COVID-19 patients

Researchers are working on causal therapy and the treatment of critical and severe manifestations downstream from the viral infection. Considering that although, theoretically complement may not be important for viral clearance in critically ill patients, it may be involved in the immunopathogenesis of SARS-CoV-2. In addition to the application of the SARS-CoV-2 vaccine, inhibiting the abnormal activation of the complement system is becoming a new research hotspot, especially in the treatment of critically ill COVID-19 patients [107, 108]. Looking for new complement inhibitors with high efficiency and low toxicity from natural products at low development cost, can provide ideas for current anti-complement therapy.

There is a strong correlation between the increased severity of COVID-19 and high concentration of complement protein [75, 107]. Compared with wild-type mice, C3-deficient mice infected with SARS-CoV exhibited less respiratory dysfunction, which was related to lower levels of cytokines, chemokines in the lungs and sera and decreased lung infiltration of neutrophils and monocytes, in spite of having an equivalent viral load in the lungs [106]. Tissue neutrophilia may be due to the neutrophil chemotaxis of complement [99]. This suggests that genetic absence of C3 and blockade of downstream complement effectors, may reduce the inflammatory lung complications of SARS-like CoVs infection [108]. Elevated concentrations of C5a and C5b-9 were detected in serum and lungs in severe cytokine storm occurred in mice infected with MERS-CoVs, suggesting the important role of C5 cleavage and excessive complement activation. The inhibition of the complement system by blocking C5a-C5a receptors can reduce MERS-CoV-mediated lung tissue damage in infected mice [109]. Therefore, complement-targeted therapy should be considered to alleviate the pathogenesis.

According to the coordinated role of C3/C5 in inflammation and coagulation, which are critical features in COVID-19, C3/C5 have become worth exploring targets. In the late stages of COVID-19, inhibiting the abnormal activation of complement (C3 and C5) can widely control ARDS, and also control the systemic inflammation affecting the microvascular bed of the kidneys, brain and other important organs in severe cases [108]. The C3 inhibitor AMY-101 was recently used safely and successfully in a severe case of COVID-19 infection, which held potential as a novel anti-inflammatory therapy [110]. Eculizumab, a humanized anti-C5 mAb, which is applied for paroxysmal nocturnal hemoglobinuria, atypical hemolytic uremia syndrome and neuromyelitis optica spectrum disorder, was used for four severe COVID-19 cases. All patients successfully recovered after treatment with Eculizumab, with a drop in inflammatory markers which indicated that Eculizumab has the potential to become a key drug for the treatment of severe COVID-19 cases [111–113].

## Natural anti-complement products provide ideas for the anti-complement therapy

Anti-complement therapy has become an effective way to control the late cytokine storm and the deterioration of the disease. Heparin is a polyanionic glycosaminoglycan, which was considered as an effective complement inhibitor in the past, but its anticoagulant effect largely limited its application [114]. Chemosynthetic complement inhibitors, such as AMY-101 and Eculizumab are too expensive for patients to afford, and drug resistance and long-term side effects require further investigation. The anti-complement components in natural compounds have low development cost and a wide range of inhibiting complement system activation effects. So far, it has been reported that polysaccharides, flavones, terpenes, steroids, saponins and other components with significant anti-complement activation have been isolated from many compounds and their microbial metabolites (See Table 1 and Additional file 1 for details). After chemical modification and in-depth mechanism research, it may provide ideas for the development of novel complement inhibition with high efficiency and low toxicity.

**Table 1:** Information of natural anti-complement products and results of in vitro and in vivo activities

Pharmacology activity	Source	In vitro anti-complementary activity	In vivo treatment of diseases associated with abnormal activation of complement
Crude Arnebiaeuchroma polysaccharides (CAEP)	*Arnebia euchroma* (Royle) I.M.Johnst	CH_50_ = 0.397 ± 0.011 mg/mL (CAEP)	CAEP treatment ameliorated febrile response and ALI induced by LPS or LPS plus ischemia reperfusion in rats, through attenuating the morphological injury, edema, and permeability in the lung and weakening the oxidant stress in BALF. CAEP treatment also improved the level of complement and complement deposition [133]
Crude polysaccharides (BCPs) Acidic polysaccharide (D3-S1)	*Bupleurum chinense* DC	CH_50_ = 0.34 ± 0.02 mg/mL AP_50_ = 0.081 ± 0.003 mg/mL (C1s, C3, C4)(D3-S1) [134]	BCPs can reduce the deposition of complement C3c in the lungs, improve pathological damage, reduce the wet-to-dry weight ratio, significantly reduce the protein concentration, white blood cell count and lung myeloperoxidase in BALF, and reduce IL-6 and TNF-α in BALF and serum to treat two-hit ALI in Wistar rats [120]. The therapeutic effect on ALI of BCPs may be related to its inhibition of the excessive activation of complement and production of pro-inflammatory mediators [121]
Crude polysaccharides (CHCP)	*Houttuynia cordata* Thunb	CH_50_ = 0.092 ± 0.020 mg/mL AP_50_ = 0.209 ± 0.036 mg/mL (C3 and C4) CHCP prevented significant macrophage migration induced by C5a and antagonized increased NO and pro-inflammatory cytokines (TNF-α, IL-6, and IL-1β) caused by LPS	CHCP inhibits inappropriate activation of the complement system and may have therapeutic implications for inflammatory diseases. CHCP significantly alleviated ALI induced by LPS. The infiltration of inflammatory cells, the expression of TLR4 and complement deposition were significantly decreased by treatment [118]. CHCP has no interference with the coagulation system [119]
Polysaccharides (JPWP) Acidic homogeneous polysaccharide isolated from JPWP (JPWP-PS) Acidic polysaccharide (YB-PS4) Water-soluble acidic polysaccharide (XB-PS3)	*Juniperus pingii var. wilsonii*	CH_50_ = 68 ± 3 μg/mL AP_50_ = 93 ± 7 μg/mL (JPWP) CH_50_ = 0.073 ± 0.009 mg/mL (JPWP-PS) [117] CH_50_ = 94.23 ± 8.9 μg/mL, AP_50_ = 194.76 ± 9.2 μg/mL (C1q, C2, C3, C4 and C5) (YB-PS4) [135] CH_50_ = 117.23 ± 18.74 μg/mL (C3, C4, C5 and C9) (XB-PS3)	JPWP significantly attenuated ALI induced by H1N1 influenza virus in vivo through reducing the inflammatory responses, alleviating oxidative stress and inhibiting the activation of complement [136]
Flavonoids-enriched extract (FESR)	*Scutellaria baicalensis* Georgi	FESR show no anti-complementary activity in vitro	FESR has great potential in treating H1N1 influenza virus-induced ALI, and the mechanism may be closely related to its antiviral, anti-inflammatory and anti-complement properties. Oral administration of FESR effectively protected infected mice, by increasing survival rate, decreasing lung index, and improving lung morphology. FESR modulated the inflammatory responses in lung tissues (TNF-α, IL-6 and MCP-1↓, IFN-γ and IL-10↑). FESR obviously reduced complement deposition and decreased complement activation product level in the lung [122]
Two new ent-labdane diterpenoids and sixteen known congeners	*Andrographis paniculata* (Burm.f.) Nees	CH_50_ and AP_50_ values of 23.1–638.3 μg/mL and 54.2–603.9 μg/mL, respectively	*Andrographis paniculata* (Burm.f.) Nees can treat various inflammatory diseases, including ALI [137], common cold and upper respiratory tract infections [138]. The ent-labdane diterpenoids obtained from it have anti-inflammatory, anti-flu and anti-cancer effects [139]
Shen-Fu Injection (SFI)	*Panax ginseng* C. A.Mey. and *Aconitum carmichaelii* Debeaux		SFI can significantly alleviate immune dysfunction after resuscitation by regulating complement expression and cytokine levels (reduce IL-6, IL-8 and TNF-α; increase IL-4 and IL-10) [123]

CH_50_ and AP_50_ stand for 50% hemolytic inhibition concentration through the classical and alternative pathway, respectively. LPS: lipopolysaccharide; BALF: bronchoalveolar lavage fluid; ALI: acute lung injury

It is encouraging that numerous polysaccharides and their sulfation products extracted from heat-clearing traditional Chinese medicines have been reported to exhibit anti-complement activity in vitro, exhibiting no or weak anticoagulant properties [115, 116]. The *Juniperus pingii* var. Wilsonii polysaccharide (JPWP) can reduce inflammatory responses, alleviate oxidative stress and inhibit the activation of complement to significantly attenuate ALI induced by H1N1 influenza virus in mice. At the same time, an acidic homogeneous polysaccharide JPWP-PS was isolated under the guidance of anti-complementary activity, and its structure was further clarified with the CH_50_ value of 0.073 ± 0.009 mg/mL [117]. JPWP-PS may be a potential compound for COVID-19 anti-complement therapy. The Crude polysaccharides isolated from *Houttuynia cordata* Thunb. (CHCP) significantly alleviated ALI induced by LPS in mice. In vivo, it was found that inflammatory cells’ infiltration, toll-like receptor 4 expression and complement deposition in the lungs of mice were significantly reduced by CHCP treatment. In vitro, CHCP prevented significant macrophage migration induced by C5a and antagonized increased NO and pro-inflammatory cytokines (TNF-α, IL-6, and IL-1β) caused by LPS. These results show that CHCP alleviates and treats LPS-induced ALI, which may be related to its inhibition of excessive activation of complement and macrophages [118]. Additionally, CHCP has no interference with the coagulation system [119]. *Bupleurum chinense* DC., a traditional Chinese medicine, has various effects like immunomodulatory, antipyretic, analgesic, anti-inflammatory and hepato-protective. The crude polysaccharides isolated from it (BCPs) can reduce the deposition of complement C3c in the lungs, improve pathological damage, reduce the wet-to-dry weight ratio, significantly reduce the protein concentration, white blood cell count and lung myeloperoxidase in bronchoalveolar lavage (BAL), and reduce IL-6 and TNF-α in BAL fluid (BALF) and serum to treat two-hit ALI in rats [120]. The therapeutic effect has also been verified in LPS-induced ALI of mice, and is speculated to be related to its inhibition of the excessive activation of complement and production of pro-inflammatory mediators [121].

A study has found that flavonoids-enriched extract from *Scutellaria baicalensis* Georgi (FESR) alleviated H1N1-induced ALI, which may be associated with the anti-complementary activity of aglycones such as oroxylin A, chrysin, wogonin and baicalein. Oral administration of FESR effectively protected H1N1 influenza virus infected mice, increasing the survival rate, decreasing the lung index, and improving the lung morphology. FESR significantly reduced the levels of complement deposition and complement activation products in the lung, but also reduced the levels of TNF-α, IL-6 and MCP-1 and increased the level of IFN-γ and IL-10 to modulate the inflammatory response of lung tissue [122]. In China, Shenfu injection (SFJ) was recommended for the treatment of severe COVID-19. Currently, a multi-center, randomized controlled, open-label clinical study of Shenfu injection for the treatment of severe COVID-19 is underway (ChiCTR2000030043). SFI can significantly alleviate immune dysfunction after ischemia accompanying cardiac arrest and resuscitation by regulating complement expression and cytokine levels [123]. This result undoubtedly provides support for the anti-complement function of SFJ and its application in critically ill COVID-19 patients.

## Insights on the development of anti-complement drugs from natural products

Although natural products with anti-complement effects in vivo and/or in vitro have been sorted out, how they reduce the complement pathway and whether they specifically target/bind complement for inhibiting complement cascade need more further experimental clarification. For potential anti-complement compounds, it is necessary to analyze their pharmacokinetic properties including absorption, distribution, metabolism, excretion, and toxicity of drugs (ADMET) through traditional pharmacokinetic study and/or virtual computer screening technology. It's worth noting that many active groups and branch structures play important roles in the anti-complement activity of natural products, which will greatly prompt the research of compound structure optimization and modification (See Additional file 1 for details). Development of preparations, chemical manufacture and control are required be improved. These products with bioavailability reaching the development level should be conducted detailed and rigorous experimental and clinical studies on patient response, therapeutic and/or toxic effects to promote the application of anti-complement strategies in critically ill COVID-19 patients.

At present, Chinese herbal prescription is still the main form of clinical use of Chinese medicine. Various prescriptions used in traditional Chinese medicine (TCM) have been suggested for treating COVID-19, the efficacy of these therapies achieved encouraging results, such as “Qingfei Paidu Tang”, “Huashi Baidu Fang” and “Xuanfei Baidu Fang” [124]. The pharmacological mechanism of Chinese herbal prescriptions is very complicated, and it emphasizes the synergistic effect of multiple components and multiple targets. Even though natural anti-complement products have advantages of low cost and wide availability, they are often used in a mixed manner in TCM. This makes it difficult to identify the specific drug agent that is effective to treat specific pathological conditions in COVID-19 therapy. Under the guidance of TCM theory and clinical efficacy, the effective multi-component Chinese medicines are the compound groups extracted from TCM, which promote the construction of optimal design model for selecting TCM prescriptions [125]. The effective multi-component Chinese medicines have the characteristics of compatibility of Chinese medicine theory, clear clinical indications and strong pertinence. The quality of effective multi-component Chinese medicines are stable and controllable, and the pharmacological basis and mechanism of action can be revealed relatively clearly at the cellular and molecular pharmacological level. This method is more able to express the inherent rules of prescription and promote the research on the pharmacological mechanism of TCM herbal prescriptions for the treatment of diseases.

From now, there are many research ideas on the effective multi-component Chinese medicines including idea based on separation and analysis, idea based on the relationship between spectrum and effect, idea based on serum pharmacochemistry and metabolomics, ideas based on biochromatography and network pharmacology. Previous studies have used phytochemical separation and pharmacological activity tracing methods to screen the effective TCM components. Compounds isolated by in vitro phytochemical methods can be screened for a variety of pharmacological activities, but whether these compounds can be absorbed into blood is still unclear. The link between the in vitro and the in vivo is lacking, which is one of the research dilemmas of natural anti-complement products from TCM and Chinese herbal prescriptions. Based on the fingerprint of TCM, the main study of pharmacodynamics is to reveal the main active ingredients or pharmacodynamic substances in TCM prescriptions. On this basis, the research model of identification and quality control of effective multi-component pharmacodynamic Chinese medicines based on the “knock-out/knock-in” of target components can be established [126]. It can clarify the relationship between fingerprint characteristics and pharmacodynamic characteristics, that determine the corresponding quality control indexes and make the constructed fingerprint more specific.

Serum pharmacochemistry of TCM suggests that the components enter into the blood after oral administration may be the effective components of TCM [127]. Combining the serum pharmacochemistry with metabolomics, liquid chromatography/mass spectrometry (LC/MS), gas chromatography/mass spectrometry (GC/MS), and nuclear magnetic resonance (NMR) techniques are applied to identify migrated components in the blood. Study the correlation between components with pharmacodynamics, and establish a biological evaluation system for the efficacy of prescriptions, so we can quickly reveal the pharmacodynamic substance basis from TCM [128]. The combination of biochromatography with various technologies, such as NMR, MS, ultraviolet, etc., is specific and selective, which can realize the simultaneous implementation of separation and activity screening [129]. The network pharmacology of TCM has the characteristics of holistic and dynamic. Through multi-target and multi-way drug analysis and evaluation, the intervention of TCM on diseases can be described from the network level [130]. However, this research strategy cannot include drug dosage into the research scope, and there is a risk of bias in literature quality. More critically, the results of network pharmacology must be experimentally verified by pharmacodynamics and pharmacological mechanism.

**Fig. 2:**
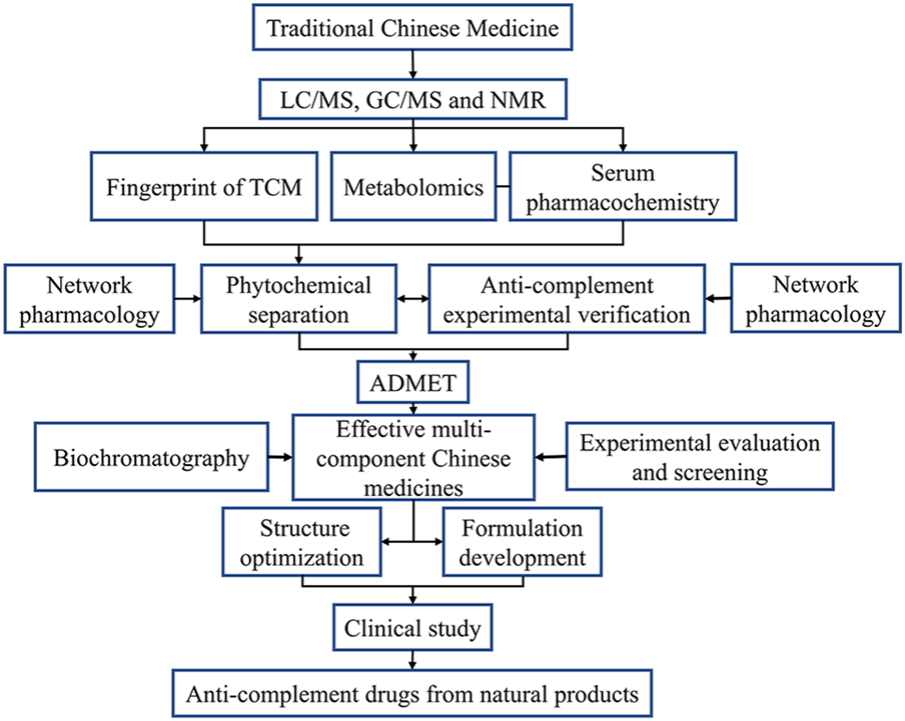
Insights on the development of anti-complement drugs from natural products. Firstly, the fingerprint of traditional Chinese medicine (TCM) can be established through LC/MS, GC/MS and NMR. Secondly, combining metabolomics and serum pharmacochemistry, the potential anti-complement medicinal ingredients are preliminarily filtered out through phytochemical separation, anti-complement experimental verification and network pharmacology. Thirdly, after the study of absorption, distribution, metabolism, excretion, and toxicity of drugs (ADMET), the effective multi-component Chinese medicines including prototype and metabolic components will be found. In this process, potential anti-complement compounds with definite efficacy and specific targets can be screened by biochromatography and experimental evaluation (in vivo and in vitro experiments). Finally, for products whose bioavailability has reached the development level, that can enter detailed and rigorous clinical studies after structure optimization and formulation development. Researches on patient response, treatment and/or toxicity is the key end-point control to promote the application of anti-complement strategies in critically ill patients with COVID-19

The pharmacological mechanism of TCM is difficult to be explained clearly due to the characteristics of multi-component, multi-target and multi-effect. Research on effective multi-component Chinese medicines has improved the reliability of verification of efficacy and pharmacological mechanism [131, 132]. In our opinion, the anti-complement components from TCM and herbal prescription can be screened and verified via the combined application of multiple methods. Through ADMET study, structure optimization and dosage form design, the application of the anti-complement drugs and clinical trials will be gradually promoted (Fig. 2).

## Conclusions

Existing clinical evidence and laboratory results have confirmed that cytokine storm is the main culprit in the deterioration of COVID-19. Especially in critically ill patients, controlling the cytokine storm has become the primary principle. The control of cytokine storm is mainly based on a variety of non-specific immunosuppressive strategies and selective cytokine blockade, which has shown a certain degree of curative effect in severe COVID-19 patients. The cytokine storm with lymphopenia in critically ill patients is particularly severe. If the storm mechanism is not effectively regulated in the upstream stage, its long-term efficacy is worrying. Complement activation, especially C3a and C5a, has been widely confirmed to be involved in the development of ALI caused by pathogenic viruses. SARS-CoV-2 infection activates coagulation and thrombotic microangiopathy by interacting with complement, pro-inflammatory cells and cytokines. It is suggested that the complement may be a promising target for the control of post-infection complications, especially cytokine storm. It is worth pondering that targeted complement inhibition therapy needs to consider the patient's constitution and disease progression, more controlled studies are needed to determine the therapeutic potential of inhibition for COVID-19 and the appropriate population.

In short, the theoretical feasibility of the application of anti-complement strategies in critically ill COVID-19 patients is gradually being recognized, and some early clinical trials have also shown gratifying results. Combining anti-complement therapy on the basis of antiviral and antibacterial drugs will maximize the therapeutic effect. Natural anti-complement ingredients seem to be a beneficial supplement to this strategy, providing a promising perspective for the development of related drugs.

## Supplementary Information

**Additional file 1.** Natural products with anti-complement activity.

## Data Availability

Not applicable.

## Abbreviations

SARS: Severe acute respiratory syndrome
MERS: Middle East respiratory syndrome
COVID-19: Severe coronavirus disease 2019
SARS-CoV-2: Severe acute respiratory syndrome coronavirus 2
ACE2: Angiotensin-converting enzyme 2
sACE2: Soluble ACE2
TMPRSS2: Transmembrane protein serine protease 2
NK cells: Natural killer cells
DCs: Dendritic cells
T and B cells: T and B lymphocytes
ICU: Intensive care unit
ARDS: Acute respiratory distress syndrome
GM-CSF: Granulocyte–macrophage colony-stimulating factor
MCP-1: Monocyte chemoattractant protein 1
TNF-α: Tumor necrosis factor-α
JAK-STAT: Janus kinase-signal transduction and transcription activator
CP: Classical pathway
LP: Lectin pathway
AP: Alternative pathway
MAC: Membrane attack complex
DIC: Intravascular coagulation
MBL: Mannan-binding lectin
MASP-2: Mannan-binding lectin serine protease 2
NETs: Neutrophil extracellular traps
BAL: Bronchoalveolar lavage
BALF: BAL fluid
ADMET: Distribution, metabolism, excretion, and toxicity of drugs
TCM: Traditional Chinese medicine
LC/MS: Liquid chromatography/mass spectrometry
GC/M: Gas chromatography/mass spectrometry
NMR: Nuclear magnetic resonance
