# Supplementary material for: Natural products provide a new perspective for anti-complement treatment of severe COVID-19: a review
Source: Chin Med. 2021 Jul 28;16:67. doi: 10.1186/s13020-021-00478-3 (PMC8318062; doi:10.1186/s13020-021-00478-3)
Supplement: Supplementary file 1 — Additional file 1. Natural products with anti-complement activity. [file 13020_2021_478_MOESM1_ESM.docx]

| **Pharmacology activity** | **Source** | **Molecular Weight（kDa）** | **Inhibition of complement activation pathways** | **Anti-complementary activity** |
| --- | --- | --- | --- | --- |
| **Polysaccharides** |  | | | |
| Acidic homogeneous polysaccharide (HD-PS-1) | Hedyotis diffusa Willd. | 194.5 | CP and AP (C3, C4) | CH_50_=0.084±0.009 mg/mL; AP_50_=0.176±0.013 mg/mL  Uronic acids are important to anti-complement activity of HD-PS-1  (Huo et al., 2020a) |
| Homogeneous polysaccharides  (AAP01-2 and AAP01-3) | *Artemisia annua* L. | 139.78 and 49.64 | CP and AP (AAP01-2 acted on C1q, C3, C4, C5 and C9 while AAP01-3 interacted with C3, C4 and C5) | CH_50_=0.360±0.020 mg/mL; AP_50_=0.547±0.033 mg/mL  (AAP01-2)  CH_50_=1.120±0.052 mg/mL; AP_50_=1.283±0.061 mg/mL  (AAP01-3)  Relatively high contents of galacturonic acid are important for anti-complement activities of the polysaccharides (Huo et al., 2020b). |
| Homogeneous polysaccharides  (EAP20-1 and EAP20-2) | Eclipta prostrata (L.) L. | 5.2 and 6.3 | CP and AP (C1q, C1r, C1s, C2, C4, C5, C7 and C9) | CH_50_=0.112±0.013 mg/mL; AP_50_=0.198±0.043 mg/mL (EAP20-1)  CH_50_=0.118±0.015 mg/mL; AP_50_=0.273±0.038 mg/mL (EAP20-2)  (Wang et al., 2017) |
| Total polysaccharides | *Ilex latifolia* Thunb. |  | CP | Hemolysis inhibition rate reached 98% at a concentration of 0.8 mg/mL (Hu et al., 2014). |
| Two sulfated derivatives (Sul-R4-2B and Sul-R4-2C) of homogeneous water-soluble polysaccharides TPSR4-2B and TPSR4-2C | *Camellia sinensis* (L.) Kuntze | 26 and 12 | CP (C1q, C1r, C1s, C2, C5 and C9) | CH_50_=5.4±0.6 μg/mL (Sul-R4-2B)  CH_50_=5.2±0.4 μg/mL (Sul-R4-2C)  (Wang et al., 2013) |
| Sulfated derivative of homogenous water-soluble polysaccharide DPSW-A  (Sul-DPSW-A) | Taraxacum  mongolicum Hand.-Mazz. | 80.813 | CP and AP (C1q, C1r, C1s and C9) | CH_50_=3.94±0.43 μg/mL; AP_50_=42.76±0.46 μg/mL  Sulfated uronic acid is essential for anti-complementary activity. (Chen et al., 2016) |
| Branched acidic polysaccharides  (PW-PS1 and PW-PS2) | *Prunella vulgaris* L. | 300 and 8 | CP and AP (PW-PS1 acted on the C1q, C3, and C9, while PW-PS2 acted on the C1q, C2, C3, C5, and C9) | CH_50_=0.28 mg/mL; AP_50_= 0.4 mg/mL (PW-PS1)  CH_50_=0.13 mg/mL; AP_50_=0.35 mg/mL (PW-PS2)  (Du et al., 2016) |
| Fucoidan derivatives | Saccharina japonica | 5.674±0.097 to 86.417±0.385 | CP and AP | CH_50_ values are in the range of 1.8 to 149.2 μg/mL, and AP_50_ values are in the range of 13.9 to 451.2 μg/mL. The sulfated and benzoylated derivatives showed better activity than fucoidan, with higher molecular weights showing the strongest activity (Liu et al., 2018). |
| Sulfated derivative of polysaccharide  (CSPS-2B-2) | Capparis spinosa L. | 8.8 | CP | CH_50_=3.5 ± 0.2 μg/mL  (Wang et al., 2012) |
| Polysaccharides  (KCA and KCW) | Kjellmaniella crsaaifolia | 153.7 and 168.7 | CP and AP | The activities of KCA and KCW reached a plateau of the classical complement pathway inhibition at a concentration of 10 μg/mL. AP_50_=4.83 and 18.6 μg/mL (KCA and KCW) (Zhang et al., 2015). |
| **Flavonoids** |  | | | |
| Apigenin-7-*O*-glucoside, apigenin-7-*O*-rutinoside, ligustroflavone, luteolin-7-*O*-glucoside and luteolin-4'-*O*-glucoside | Ligustrum vulgare L. and Phillyrea latifolia L. |  | CP | IC_50_=52.5, 18.1, 86.8, 15.8, 45.1 and 67.2 μM (Pieroni et al., 2000). |
| Metabolites of flavonoid aglycones (baicalein, wogonin, chrysin and oroxylinA) | *Scutellaria baicalensis* Georgi |  | CP and AP | Flavonoids-enriched extract has a great potential for the treatment of ALI induced by H1N1 virus with antiviral, anti-inflammatory and anti-complementary properties. CH_50_ values are in the range of 0.26±0.01 to 0.76±0.01 mg/mL, and AP_50_ value is in the range of 0.43±0.01 to 0.91±0.02 mg/mL (metabolites of flavonoid aglycones) (Zhi et al., 2019). |
| Acetylated flavonoid glycosides | Centaurium spicatum (L.) Fritsch |  | CP | IC_50_ values are in the range of 10±0.9 to 59.3 μM  (Shahat et al., 2003). |
| Flavonoids Afzelin and quercitrin;  Galloyl glucoses 1,2,6-trigalloylglucose and 1,2,3,6-tetragalloylglucose | *Juglans mandshurica* Maxim. |  | CP | IC_50_=258, 440, 136 and 34 μM (Min et al., 2003). |
| Total flavonoids | Penthorum sedoides var. chinense (Pursh) Maxim. |  | CP | IC_50_=111.6 µg/ml  (Zeng et al., 2013) |
| Flavonoids and their metabolites | *Sophora tonkinensis* Gagnep. |  | CP and AP | CH_50_ values range from 0.082 ± 0.029 to 3.517±1.367 mg/mL, while AP_50_ values range from 0.217 ± 0.068 to 0.307±0.089 mg/mL. Some glycosides are metabolized into aglycones with more potent anticomplement activity (Jin et al., 2020). |
| Metabolites of grosvenorine | *Thladiantha grosvenorii* (Swingle) C.Jeffrey |  | CP and AP | Grosvenorine does not show any obvious anticomplement activity, while its four metabolites exhibite significant activities.  CH_50_=17.0±5.2 mg/mL (kaempferol); AP_50_=125.5±27.1 mg/mL (a-rhamnoisorobin); AP_50_=172.0±21.2 mg/mL (afzelin); AP_50_=29.1±7.4 mg/mL (kaempferol).  The number of free OH groups on the A-ring of 5,7-dihydroxyflavone, presence of the 3-OH group, glycosylation of 3-OH and 7-OH and free OH position may affect the anti-complement activity of flavonols (Wang et al., 2015). |
| Flavonoids (kaempferol-3-Orutinoside and kaempferol) | Chimonanthus nitens Oliv. |  | CP | CH_50_=0.053±0.013 and 0.041±0.036 mg/mL  (kaempferol-3-Orutinoside and kaempferol)(Huang et al., 2020). |
| Flavonoids | Litsea japonica (Thunb.) Juss. | 433, 449 and 595(Afzelin, quercitrin and tiliroside) | CP | IC_50_=258, 440, and 101 µM (Afzelin, quercitrin and tiliroside).  The inhibitory potencies of flavonoids and aglycones against complement activity increase in inverse proportion to number of free hydroxyls on B-ring of 5,7-dihydroxyflavone (Lee et al., 2005). |
| **Terpenoids** |  | | | |
| Diterpenoids | Clerodendrum bungei Steud*.* |  | CP | Five diterpenoids named 12-O-β-d-glucopyranosyl-3,11,16-trihydroxyabieta-8,11,13-triene, 3,12-O-β-d-diglucopyranosyl-11,16-dihydroxyabieta-8,11,13-triene, ajugaside A, uncinatone and 19-hydroxyteuvincenone F, showed inhibitory activity against complement system with IC_50_ values of 24, 138, 116, 87 and 232 µM (Kim et al., 2010). |
| Triterpenoid (Podocarpasid) | *Actaea podocarpa* DC. |  | CP | IC_50_=190 µM (Ali et al., 2006). |
| Triterpenoids | *Aceriphyllum rossii* (Oliv.) Engl. |  | CP | 3-oxoolean-12-en-27-oic acid, 3α-hydroxyolean-12-en-27-oic acid and 3α,23-diacetoxyolean-12-en-27-oic acid show significant anticomplement activity on the CP with IC_50_ values of 71.4, 98.5, and 180.7 μM respectively. Both the ketone at C-3 and the methyl at C-23 in the oleanane triterpenoids with a carboxyl group at C-27 are important for the anticomplement activity against the CP (Min et al., 2008). |
| Triterpenoids | *Ilex asprella* (Hook. & Arn.) Champ. ex Benth. |  | CP and AP (The targets of several bioactive triterpenoids in complement activation cascade were identified.) | Several triterpenoids (1–3, 6–7) exhibited potent anticomplement activity with the CH_50_ and AP_50_ values of 0.058–0.131 mg/mL and 0.080–0.444 mg/mL, respectively. The ursane and oleanane triterpenoids are found to show anticomplement activity with some structure-activity relationships. Caffeoyl group can enhance activity remarkably, followed by coumaroyl and feruloyl group. The 28-carboxyl group is also important to anticomplement activity for the triterpenoids (Wen et al., 2017). |
| Triterpenoids and other constituents | Benincasa hispida (Thunb.) Cogn. |  | CP | CP_50_ values range from 115.6±10.4 to 282.4±27.1 μM.  (Han et al., 2013) |
| Oleanane-type triterpenes  (3-*O*-*cis*-pcoumaroyl maslinic acid, 3-*O*-*trans*-*p*-coumaroyl maslinic acid and oleanolic acid) | *Ziziphus jujuba* Mill. |  | CP | IC_50_=101.4, 143.9, and 163.4 μM (Lee et al., 2004). |
| New 9,19-cycloartane triterpene | Beesia calthifolia Ulbr. | 819.4525 | CP | New 9,19-cycloartane triterpene showed moderately anticomplement activity, similar to that of rosmarinic acid (IC50 value 181.8 µM). Compounds 3 and 4 showed weak active (IC50 value 467.4 ± 23.1 and 363.0 ± 9.1 µM) and 5 was inactive（three cycloartane glycosides (3–5). OH at C-12, C-18 and C-15 along with the polarity could affect the inhibitory activity (Zhao et al., 2016). 6'-*O*-(4''-hydroxy-3''-methoxy-benzoyl)-β-_D_-glucosyl of 9,19-cycloartane-type triterpenoids is essential for anticomplement activity (Mu et al., 2014). |
| Oleanane-type Triterpenes | *Aceriphyllum rossii* (Oliv.) Engl. |  | CP | Compounds 1, 2 and 5 showed anticomplement activities, with IC50 values of 328.4, 77.5 and 348.6 µM, respectively. A carboxyl group at the C-23 position and an α-configuration of a hydroxyl group at the C-3 position in the olean-29-carboxylic acid triterpenoids seem to play important roles in the anticomplement activity (Min, 2012). |
| Cycloartane-type triterpene  glycosides | *Cimicifuga heracleifolia* Kom. | 689.6 and 703.4 | CP | IC_50_=7.7 µM  ((23R,24R)-25-O-acetylcimigenol 3-O-β-D-xylopyranoside)  IC_50_=195.6 µM  (24-epi-24-O-acetylhydroshengmanol 3-O-β-D-xylopyranoside)  (Lee et al., 2012). |
| **Steroids** |  |  |  |  |
| Citrostadienol | Schisandra chinensis (Turcz.) Baill. | 426 | CP | IC_50_=4.6*10^-8^ M (Lee et al., 1997). |
| Steroids (ergosterol peroxide and ergosterol) | Ganoderma lucidum (Fr.) Karst. | 428 and 396 | CP | IC_50_=126.8 and 52.0 µM (Seo et al., 2009). |
| New polyhydroxylated pregnane (lβ,2β,3β,4β,5β,6β-hexolhydroxy-pregn-16-en-20-one) and aglycones of steroidal saponins | *Reineckia triandra* H.Karst. |  | CP | CH_50_=0.043 mg/mL.  (lβ,2β,3β,4β,5β,6β-hexolhydroxy-pregn-16-en-20-one )  Steroidal saponins showed no inhibition of anti-complement, but hydrolysis of them resulted in its aglycones correspondingly which showed anticomplement activity with the CH_50_ values of 0.049–0.156 mg/mL (Xu et al., 2019). |
| Phytosterols | Cucurbita moschata Duchesne |  | CP | IC_50_=0.74 mg/mL (Yang et al., 2002). |
| **Saponins** |  |  |  |  |
| Tetranor-cycloartane glycoside (cimilactone A) | *Cimicifuga foetida* L. |  | CP | IC_50_=28.6 μM (Qiu et al., 2006). |
| Oleanolic acid glycosides | Achyranthes japonica (Miq.) Nakai |  | CP | Achyranthoside C dimethyl ester showed the most potent inhibitory activity (IC_50_=26.2 μg/mL) (Jung et al., 2012b). |
| Monoterpenoid glucosides | *Paeonia × suffruticosa* Andrews |  | CP and AP (Suffrupaeoniflorin A interacted with C1q, C3, C5, and C9,while galloylpaeoniflorin and galloyloxypaeoniflorin acted on C1q, C3, and C5.） | The monoterpenoid glucosides 1, 2, 7, 10−19, and 22 exhibited anticomplement effects with CH_50_ and AP_50_ values ranging from 0.14 to 2.67 mM and 0.25 to 3.67 mM, respectively (Song et al., 2014). |
| Ginseng saponins | Panax ginseng C.A.Mey. |  | CP (C1q, C2, C3, C4, and C5) | Compound 2, 10, 11 and 12 were more active in this assay system, with CP_50_ values of 160, 150, 108, and 112 μM (Gao et al., 2013). |
| Lupane-Type Triterpenoidal Saponins | Pulsatilla chinensis (Bunge) Regel |  | CP | CP_50_ value range from 207.8±13.4 to 207.8±13.4 μM.  (Xu et al., 2013) |
| Glycosides | *Urtica fissa* E. Pritz. |  | CP | Some terpenoids (1, 2) and flavanoids (10, 13) possessed anticomplement activities (CP_50_ and AP_50_ values less than 100 μM) (Wang et al., 2018). |
| **Others** |  | | | |
| Polyacetylenes | *Dendropanax morbiferus* H.Lév. |  | CP | Three polyacetylenes named (3S)-falcarinol, (3S,8S)-falcarindiol and (3S)-diynene show anti-complement activity with IC_50_ values of 87.3, 15.2 and 39.8 mM (Chung et al., 2011c). The IC_50_ values of (9Z,16S)-16-hydroxy-9,17-Octadecadiene-12,14-diynoic acid is 56.98 μM (Park et al., 2004). |
| Galloyl derivatives | Quercus faginea Lam. |  | CP | 6'-O-galloyl salidroside, methyl gallate, 1,2,3,6-tetragalloylglucose, and 1,2,6-trigalloylglucose showed inhibitory activity with IC_50_ values of 224, 362.4, 32.3, and 138.3 μM (Chung et al., 2011a). |
| Oryzafuran | Black coloured rice bran |  | CP | IC_50_=126.2 μg/mL (Moon et al., 2011). |
| Quassinoids | *Brucea javanica* (L.) Merr. |  | CP and AP | Compounds 1–6 exhibited potent anticomplement activity with CH_50_ and AP_50_ values of 0.032–0.075 mg/mL and 0.061–0.118 mg/mL, respectively. Hydroxylation at C-20 in quassinoids is important for anti-complement activity (Zhan et al., 2020). |
| Phenyldilactones (1, 2), two coumarins (3, 4) and a dimer of N-E-feruloyl tyramine (5) together with twenty-three known compounds (6-28) | Polygonum chinense  L. |  | Anti-complement compounds have diverse targets in complement activation cascade. | CH_50_ and AP_50_ values ranging from 0.18 to 1.45 mM, and 0.26 to 2.80 mM, respectively (Zheng et al., 2018). |
| Ezoartemin and yamayomoginin | Artemisia montana (Nakai) Pamp. |  | CP and AP | IC_50_=251.2±48.3 μM (Ezoartemin).  IC_50_=173.2±25.3 μM (Yamayomoginin) (Moon et al., 2012). |
| Chal Sorghum ethyl acetate extract | Sorghum bicolor (L.) Moench |  | CP | IC_50_=38.7 μg/mL (Chung et al., 2011b). |
| Chloroform extract | Amarantaceae plants |  | CP | IC_50_=73.1 μg/mL (Jung et al., 2012a). |
| Xanthones Garcinone E and 1-Isomangostanin | *Mangostana garcinia* Gaertn. |  | CP | IC_50_=110.8±11.7 and 32.4±9.3 μM (Quan et al., 2010). |
| Stereoisomers of furan derivatives | Phellinus linteus (Berk et Curt) Aoshima | 307 | CP | IC_50_=33.6 and 33.7 μM (phellinusfurans A and B) (Min et al., 2006). |
| Flavonoid (5-hydroxyl-3', 4', 6, 7-tetramethoxy flavone)  Lignan (medioresinol)  Alkaloid (5-hydroxypyrrolidin-2-one) | Anchusa italica Retz. |  | CP | CH_50_ values range from 0.07 to 0.82 mM (Hou et al., 2017). |

Inhibitory activity against complement system with 50% inhibitory concentrations (IC50) values.

CH_50_ and AP_50_ stand for 50% hemolytic inhibition concentration through the classical and alternative pathway, respectively.

REFERENCES

Ali, Z., Khan, S.I., Ferreira, D., and Khan, I.A. (2006). Podocarpaside, a triterpenoid possessing a new backbone from Actaea podocarpa. *Organic Letters* 8(24)**,** 5529-5532. doi: 10.1021/ol062212s.

Chen, M.M., Wu, J.J., Shi, S.S., Chen, Y.L., Wang, H.J., Fan, H.W., et al. (2016). Structure analysis of a heteropolysaccharide from Taraxacum mongolicum Hand.-Matz. and anticomplementary activity of its sulfated derivatives. *Carbohydrate Polymers* 152**,** 241-252. doi: 10.1016/j.carbpol.2016.06.110.

Chung, I.M., Kim, E.H., Kim, J.J., and Moon, H.I. (2011a). Inhibition effects of the classical pathway complement of isolated compounds from Quercus glauca. *Human & Experimental Toxicology* 30(9)**,** 1415-1419. doi: 10.1177/0960327110390067.

Chung, I.M., Kim, M.J., Park, D.S., and Moon, H.I. (2011b). Inhibition effects of the classical pathway complement of three Sorghum bicolor from South Korea. *Immunopharmacology and Immunotoxicology* 33(3)**,** 447-449. doi: 10.3109/08923973.2010.532804.

Chung, I.M., Song, H.K., Kim, S.J., and Moon, H.I. (2011c). Anticomplement activity of polyacetylenes from leaves of Dendropanax morbifera Leveille. *Phytotherapy Research* 25(5)**,** 784-786. doi: 10.1002/ptr.3336.

Du, D.S., Lu, Y., Cheng, Z.H., and Chen, D.F. (2016). Structure characterization of two novel polysaccharides isolated from the spikes of Prunella vulgaris and their anticomplement activities. *Journal of Ethnopharmacology* 193**,** 345-353. doi: 10.1016/j.jep.2016.08.034.

Gao, H.W., Zhang, M.M., Liu, Y.L., Xu, Q.M., and Yang, S.L. (2013). Anticomplement activity of ginsenosides from Panax ginseng. *Journal of Functional Foods* 5(1)**,** 498-502. doi: 10.1016/j.jff.2012.09.007.

Han, X.N., Liu, C.Y., Liu, Y.L., Xu, Q.M., Li, X.R., and Yang, S.L. (2013). New triterpenoids and other constituents from the fruits of Benincasa hispida (Thunb.) Cogn.. *Journal of Agricultural and Food Chemistry* 61(51)**,** 12692-12699. doi: 10.1021/jf405384r.

Hou, Y.Z., Chen, K.K., Deng, X.L., Fu, Z.L., Chen, D.F., and Wang, Q. (2017). Anti-complementary constituents of Anchusa italica. *Natural Product Research* 31(21)**,** 2572-2574. doi: 10.1080/14786419.2017.1320789.

Hu, T., He, X.W., Jiang, J.G., and Xu, X.L. (2014). Efficacy evaluation of a Chinese bitter tea (Ilex latifolia Thunb.) via analyses of its main components. *Food & Function* 5(5)**,** 876-881. doi: 10.1039/c3fo60603a.

Huang, W., Wen, Z., Wang, M., Xu, B., Zhou, B., and Li, X. (2020). Anticomplement and antitussive activities of major compound extracted from Chimonanthus nitens Oliv. leaf. *Biomed Chromatogr* 34(2)**,** e4736. doi: 10.1002/bmc.4736.

Huo, J.Y., Lu, Y., Jiao, Y.K., and Chen, D.F. (2020a). Structural characterization and anticomplement activity of an acidic polysaccharide from Hedyotis diffusa. *International Journal of Biological Macromolecules* 155**,** 1553-1560. doi: 10.1016/j.ijbiomac.2019.11.132.

Huo, J.Y., Lu, Y., Xia, L., and Chen, D.F. (2020b). Structural characterization and anticomplement activities of three acidic homogeneous polysaccharides from Artemisia annua. *Journal of Ethnopharmacology* 247. doi: UNSP 11228110.1016/j.jep.2019.112281.

Jin, X., Lu, Y., Chen, S., and Chen, D. (2020). UPLC-MS identification and anticomplement activity of the metabolites of Sophora tonkinensis flavonoids treated with human intestinal bacteria. *J Pharm Biomed Anal* 184**,** 113176. doi: 10.1016/j.jpba.2020.113176.

Jung, S., Lee, J.H., Lee, Y.C., and Moon, H.I. (2012a). Anticomplement activity of organic solvent extracts from Korea local Amarantaceae spp. *Immunopharmacology and Immunotoxicology* 34(2)**,** 210-212. doi: 10.3109/08923973.2011.593180.

Jung, S., Lee, J.H., Lee, Y.C., and Moon, H.I. (2012b). Inhibitory effects of three oleanolic acid glycosides from Achyranthes japonica on the complement classical pathway. *Immunopharmacology and Immunotoxicology* 34(2)**,** 213-215. doi: 10.3109/08923973.2011.594954.

Kim, S.K., Cho, S.B., and Moon, H.I. (2010). Anti-complement activity of isolated compounds from the roots of clerodendrum bungei steud. *Phytotherapy Research* 24(11)**,** 1720-1723. doi: 10.1002/ptr.3254.

Lee, I.S., Oh, S.R., Jung, K.Y., and kkd, e. (1997). Anticomplementary activity and complete 13c nmr assignment of citrostadienol from schizandra chinensis. *Pharmaceutical Biology* 35**,** 358-363.

Lee, J.H., Cuong, T.D., Kwack, S.J., Seok, J.H., Lee, J.K., Jeong, J.Y., et al. (2012). Cycloartane-type triterpene glycosides from the rhizomes of Cimicifuga heracleifolia and their anticomplementary activity. *Planta Medica* 78(12)**,** 1391-1394. doi: 10.1055/s-0032-1314980.

Lee, S.M., Park, J.G., Lee, Y.H., Lee, C.G., Min, B.S., Kim, J.H., et al. (2004). Anti-complementary activity of triterpenoides from fruits of Zizyphus jujuba. *Biological & Pharmaceutical Bulletin* 27(11)**,** 1883-1886. doi: DOI 10.1248/bpb.27.1883.

Lee, S.Y., Min, B.S., Kim, J.H., Lee, J., Kim, T.J., Kim, C.S., et al. (2005). Flavonoids from the leaves of Litsea japonica and their anti-complement activity. *Phytotherapy Research* 19(4)**,** 273-276. doi: 10.1002/ptr.1453.

Liu, H.D., Wang, J., Zhang, Q.B., and Zhang, H. (2018). The effect of different substitute groups and molecular weights of fucoidan on neuroprotective and anticomplement activity. *International Journal of Biological Macromolecules* 113**,** 82-89. doi: 10.1016/j.ijbiomac.2018.02.109.

Min, B.S. (2012). Anticomplementary activity of oleanane-type triterpenes from the roots of Aceriphyllum rossii. *Archives of Pharmacal Research* 35(6)**,** 1003-1008. doi: 10.1007/s12272-012-0607-8.

Min, B.S., Lee, I., Chang, M.J., Yoo, J.K., Na, M., Hung, T.M., et al. (2008). Anticomplementary activity of triterpenoids from the whole plant of Aceriphyllum rossii against the classical pathway. *Planta Medica* 74(7)**,** 726-729. doi: 10.1055/s-2008-1074534.

Min, B.S., Lee, S.Y., Kim, J.H., Lee, J.K., Kim, T.J., Kim, D.H., et al. (2003). Anti-complement activity of constituents from the stem-bark of Juglans mandshurica. *Biol Pharm Bull* 26(7)**,** 1042-1044. doi: 10.1248/bpb.26.1042.

Min, B.S., Yun, B.S., Lee, H.K., Jung, H.J., Jung, H.A., and Choi, J.S. (2006). Two novel furan derivatives from Phellinus linteus with anti-complement activity. *Bioorganic & Medicinal Chemistry Letters* 16(12)**,** 3255-3257. doi: 10.1016/j.bmcl.2006.03.027.

Moon, H.I., Jung, S., Lee, Y.C., and Lee, J.H. (2012). Anticomplement activity of isolated compounds from Artemisia montana. *Immunopharmacology and Immunotoxicology* 34(1)**,** 113-115. doi: 10.3109/08923973.2011.583924.

Moon, H.I., Lee, J.H., Lee, Y.C., and Kim, S.K. (2011). Inhibitory effects of isolated compounds from black coloured rice bran on the complement classical pathway. *Phytotherapy Research* 25(9)**,** 1418-1420. doi: 10.1002/ptr.3485.

Mu, L.H., Li, H.J., Guo, D.H., Zhao, J.Y., and Liu, P. (2014). Cycloartane-type triterpene glycosides from Beesia calthaefolia and their anticomplement activity. *Journal of Natural Medicines* 68(3)**,** 604-609. doi: 10.1007/s11418-014-0820-5.

Ou, Y.Y., Jiang, Y., Li, H., Zhang, Y.Y., Lu, Y., and Chen, D.F. (2017). Polysaccharides from Arnebia euchroma ameliorated endotoxic fever and acute lung injury in rats through inhibiting complement system. *Inflammation* 40(1)**,** 275-284. doi: 10.1007/s10753-016-0478-0.

Park, B.Y., Min, B.S., Oh, S.R., Kim, J.H., Kim, T.J., Kim, D.H., et al. (2004). Isolation and anticomplement activity of compounds from Dendropanax morbifera. *Journal of Ethnopharmacology* 90(2-3)**,** 403-408. doi: 10.1016/j.jep.2003.11.002.

Pieroni, A., Pachaly, P., Huang, Y., Van Poel, B., and Vlietinck, A.J. (2000). Studies on anti-complementary activity of extracts and isolated flavones from Ligustrum vulgare and Phillyrea latifolia leaves (Oleaceae). *J Ethnopharmacol* 70(3)**,** 213-217. doi: 10.1016/s0378-8741(99)00169-5.

Qiu, M.H., Kim, J.H., Lee, H.K., and Min, B.S. (2006). Anticomplement activity of cycloartane glycosides from the rhizome of Cimicifuga foetida. *Phytotherapy Research* 20(11)**,** 945-948. doi: 10.1002/ptr.1982.

Quan, G.H., Oh, S.R., Kim, J.H., Lee, H.K., Kinghorn, A.D., and Chin, Y.W. (2010). Xanthone constituents of the fruits of Garcinia mangostana with anticomplement activity. *Phytotherapy Research* 24(10)**,** 1575-1577. doi: 10.1002/ptr.3177.

Seo, H.W., Hung, T.M., Na, M., Jung, H.J., Kim, J.C., Choi, J.S., et al. (2009). Steroids and triterpenes from the fruit bodies of Ganoderma lucidum and their anti-complement activity. *Archives of Pharmacal Research* 32(11)**,** 1573-1579. doi: 10.1007/s12272-009-2109-x.

Shahat, A.A., Cos, P., Hermans, N., Apers, S., De Bruyne, T., Pieters, L., et al. (2003). Anticomplement and antioxidant activities of new acetylated flavonoid glycosides from Centaurium spicatum. *Planta Med* 69(12)**,** 1153-1156. doi: 10.1055/s-2003-818009.

Song, W.H., Cheng, Z.H., and Chen, D.F. (2014). Anticomplement monoterpenoid glucosides from the root bark of Paeonia suffruticosa. *Journal of Natural Products* 77(1)**,** 42-48. doi: 10.1021/np400571x.

Wang, H.J., Shi, S.S., Gu, X.L., Zhu, C., Wei, G.D., Wang, H.W., et al. (2013). Homogalacturonans from preinfused green tea: structural characterization and anticomplementary activity of their sulfated derivatives. *Journal of Agricultural and Food Chemistry* 61(46)**,** 10971-10980. doi: 10.1021/jf401947n.

Wang, H.J., Wang, H.W., Shi, S.S., Duan, J.Y., and Wang, S.C. (2012). Structural characterization of a homogalacturonan from Capparis spinosa L. fruits and anti-complement activity of its sulfated derivative. *Glycoconjugate Journal* 29(5-6)**,** 379-387. doi: 10.1007/s10719-012-9418-x.

Wang, H.W., Li, N., Zhu, C., Shi, S.S., Jin, H., and Wang, S.C. (2017). Anti-complementary activity of two homogeneous polysaccharides from Eclipta prostrata. *Biochemical and Biophysical Research Communications* 493(2)**,** 887-893. doi: 10.1016/j.bbrc.2017.09.126.

Wang, M., Xing, S., Luu, T., Fan, M., and Li, X. (2015). The gastrointestinal tract metabolism and pharmacological activities of grosvenorine, a major and characteristic flavonoid in the fruits of Siraitia grosvenorii. *Chem Biodivers* 12(11)**,** 1652-1664. doi: 10.1002/cbdv.201400397.

Wang, M.Y., Zhang, Y., Zhang, H., Feng, X.R., and Li, X.B. (2018). The active glycosides from Urtica fissa rhizome decoction. *Journal of Natural Medicines* 72(2)**,** 557-562. doi: 10.1007/s11418-018-1172-3.

Wen, Q., Jin, X., Lu, Y., and Chen, D.F. (2020). Anticomplement ent-labdane diterpenoids from the aerial parts of Andrographis paniculata. *Fitoterapia* 142. doi: ARTN 10452810.1016/j.fitote.2020.104528.

Wen, Q., Lu, Y., Chao, Z., and Chen, D.F. (2017). Anticomplement triterpenoids from the roots of Ilex asprella. *Bioorganic & Medicinal Chemistry Letters* 27(4)**,** 880-886. doi: 10.1016/j.bmcl.2017.01.007.

Xia, L., Deji, Zhu, M.X., Chen, D.F., and Lu, Y. (2020). Juniperus pingii var. wilsonii acidic polysaccharide: Extraction, characterization and anticomplement activity. *Carbohydrate Polymers* 231. doi: ARTN 11572810.1016/j.carbpol.2019.115728.

Xia, L., Li, B.B., Lu, Y., and Chen, D.F. (2019). Structural characterization and anticomplement activity of an acidic polysaccharide containing 3-O-methyl galactose from Juniperus tibetica. *International Journal of Biological Macromolecules* 132**,** 1244-1251. doi: 10.1016/j.ijbiomac.2019.04.029.

Xie, J.Y., Di, H.Y., Li, H., Cheng, X.Q., Zhang, Y.Y., and Chen, D.F. (2012). Bupleurum chinense DC polysaccharides attenuates lipopolysaccharide-induced acute lung injury in mice. *Phytomedicine* 19(2)**,** 130-137. doi: 10.1016/j.phymed.2011.08.057.

Xu, Q.M., Shu, Z., Zhu, W.F., Liu, Y.L., Li, X.R., and Yang, S.L. (2013). Lupane-type triterpenoidal saponins from Pulsatilla chinensis and their anticomplement activities through the classical pathway. *Planta Medica* 79(6)**,** 506-512. doi: 10.1055/s-0032-1328323.

Xu, X., Wu, B., Zhan, Y.Z., Huang, W.P., Yang, S.L., Wen, Q., et al. (2019). Steroids from herbs of Reineckia carnea and their anticomplement activities. *Natural Product Research* 33(11)**,** 1570-1576. doi: 10.1080/14786419.2017.1423309.

Yang, J.O., Oh, S.R., Lee, H.K., Kim, C.J., and Song, K.B. (2002). Isolation of anticomplementary substances from Cucurbita moschata Duch. *Journal of Food Science* 67(4)**,** 1348-1351. doi: DOI 10.1111/j.1365-2621.2002.tb10286.x.

Zeng, Q.H., Zhang, X.W., Xu, X.L., Jiang, M.H., Xu, K.P., Piao, J.H., et al. (2013). Antioxidant and anticomplement functions of flavonoids extracted from Penthorum chinense Pursh. *Food Funct* 4(12)**,** 1811-1818. doi: 10.1039/c3fo60342c.

Zhan, Y.Z., Tan, T., Qian, K., Yang, S.L., Feng, Y.L., and Wen, Q. (2020). Quassinoids from seeds of Brucea Javanica and their anticomplement activities. *Natural Product Research* 34(8)**,** 1186-1191. doi: 10.1080/14786419.2018.1550764.

Zhang, Q., Li, C.S., Wang, S., and Gu, W. (2016). Effects of Chinese medicine Shen-Fu Injection on the expression of inflammatory cytokines and complements during post-resuscitation immune dysfunction in a porcine model. *Chinese Journal of Integrative Medicine* 22(2)**,** 101-109. doi: 10.1007/s11655-014-1857-8.

Zhang, W.J., Jin, W.H., Sun, D.L., Zhao, L.Y., Wang, J., Duan, D.L., et al. (2015). Structural analysis and anti-complement activity of polysaccharides from Kjellmaniella crsaaifolia. *Marine Drugs* 13(3)**,** 1360-1374. doi: 10.3390/md13031360.

Zhao, J.Y., Mu, L.H., Dong, X.Z., Hu, Y., and Liu, P. (2016). One new cycloartane triterpene glycoside from Beesia calthaefolia. *Natural Product Research* 30(3)**,** 316-321. doi: 10.1080/14786419.2015.1058791.

Zheng, H.C., Lu, Y., and Chen, D.F. (2018). Anticomplement compounds from Polygonum chinense. *Bioorganic & Medicinal Chemistry Letters* 28(9)**,** 1495-1500. doi: 10.1016/j.bmcl.2018.03.079.

Zhi, H.J., Zhu, H.Y., Zhang, Y.Y., Lu, Y., Li, H., and Chen, D.F. (2019). In vivo effect of quantified flavonoids-enriched extract of Scutellaria baicalensis root on acute lung injury induced by influenza A virus. *Phytomedicine* 57**,** 105-116. doi: 10.1016/j.phymed.2018.12.009.

Zhi, H.W., Zhang, Y.Y., Zhang, J.W., and Chen, D.F. (2008). Isolation and characterization of an anti-complementary protein-bound polysaccharide from the stem barks of Eucommia ulmoides. *International Immunopharmacology* 8(9)**,** 1222-1230. doi: 10.1016/j.intimp.2008.04.012.
